# Transport time as a potential limiting factor for thrombolytic treatment of stroke in Norway

**DOI:** 10.1186/s12913-025-12503-4

**Published:** 2025-03-14

**Authors:** Jørgen Ibsen, Maren Ranhoff Hov, Torunn Varmdal, Christian Georg Lund, Christian Hall

**Affiliations:** 1 Medical Department, Ringerike Hospital, Vestre Viken Hospital Trust, Box 800, Hønefoss, Drammen, 3004 Norway; 2 The Norwegian Air Ambulance Foundation, Oslo, Norway; 3 Bachelor of Paramedic, Oslo Metropolitan University, Oslo, Norway; 4 Department of Neurology, Oslo University Hospital, Oslo, Norway; 5 Department of Circulation and Medical Imaging, Norwegian University of Science and Technology, Trondheim, Norway; 6 Department of Medical Quality Registries, St. Olav´s Hospital, Trondheim, Norway; 7 Institute of Clinical Medicine, University of Oslo, Oslo, Norway

**Keywords:** Health-registry, Computed tomography, Thrombolysis, Rural, Outcome

## Abstract

**Background:**

Rapid diagnosis and treatment of stroke is important for good outcome. In some cases, patients with ischemic stroke arrive at hospital too late for reperfusion therapy. This may be the case especially in rural areas where time spent during transport may delay or even preclude thrombolytic treatment after hospital arrival. We aimed to estimate the extent and clinical relevance of this problem in the Norwegian population.

**Methods:**

We collected data for all reported acute ischemic strokes for the years 2017 and 2018. Transport times from home to hospital were calculated from geographical data and related to frequency of thrombolysis, thrombectomy and clinical outcome after 3 months.

**Results:**

The frequency of reperfusion therapy decreased significantly as transport time increased. Six percent (564) of 9 428 patients were classified as having a prolonged time in transport. In this group, frequency of intravenous thrombolysis was 10,5% as opposed to 28,2% when prolonged transport was not present. Thrombectomy was performed in 2.1% versus 4.9% in the two groups respectively. We did not find a statistically significant difference between the two groups with regard to clinical outcome as judged by the modified Rankin Scale.

**Conclusion:**

In the years 2017 and 2018 a relatively small group of Norwegian patients with prolonged time in transport was disfavored concerning access to reperfusion therapy for ischemic stroke. In such cases a prehospital solution for diagnostic work up and treatment might improve access to acute stroke treatment.

## Introduction

According to the National Stroke Registry approximately 10 000 Norwegian patients suffer a stroke annually [1]. The effect of thrombolytic treatment for ischemic stroke is time dependent [2–4]. Accordingly, national recommendations state that such treatment should be administered within 3 h after symptoms onset and no later than 4.5 h [5]. Advanced neuroradiological examination may expand this time window for patients with stroke of unknown onset or “wake up stroke”, albeit only for a minor proportion of patients and at a higher risk of symptomatic intracranial hemorrhage [6]. Patients with Large or Medium Vessel Occlusions may benefit from intraarterial thrombectomy [7, 8]. This procedure can be effective when performed up to 24 h after onset of stroke symptoms [9, 10].

The delay from onset of symptoms to thrombolytic treatment can be separated into several components: Time from onset of symptoms to alarm (“patients delay”), time from alarm to ambulance arrival, ambulance on scene time, transport time to hospital and time from arrival at the emergency room to treatment (“door to needle time”). Of these time components, time spent during ambulance transport to the nearest Computed Tomography (CT) equipped hospital, sometimes on suboptimal road standard, may put Norwegian patients living in rural districts at a disadvantage with regard to access to thrombolysis [11, 12].

The Norwegian Health Authorities aim to offer equal access to health services for all inhabitants [13]. However, due to the geographical heterogeneity of Norway with relatively large rural districts, there is reason to suspect an uneven access to acute stroke care. The degree to which transport time is a limiting factor for thrombolytic treatment in Norway has not been reported.

In the present study we analyzed registry data for Norwegian stroke patients over a 2-year period. Assuming symptom onset at home we determined the relation between estimated time spent in transport, frequency of reperfusion therapy and clinical outcome 3 months after stroke. We hypothesized that estimated transport time would be inversely related to frequency of reperfusion therapy as well as stroke outcome.

## Materials and methods

**Fig. 1:**
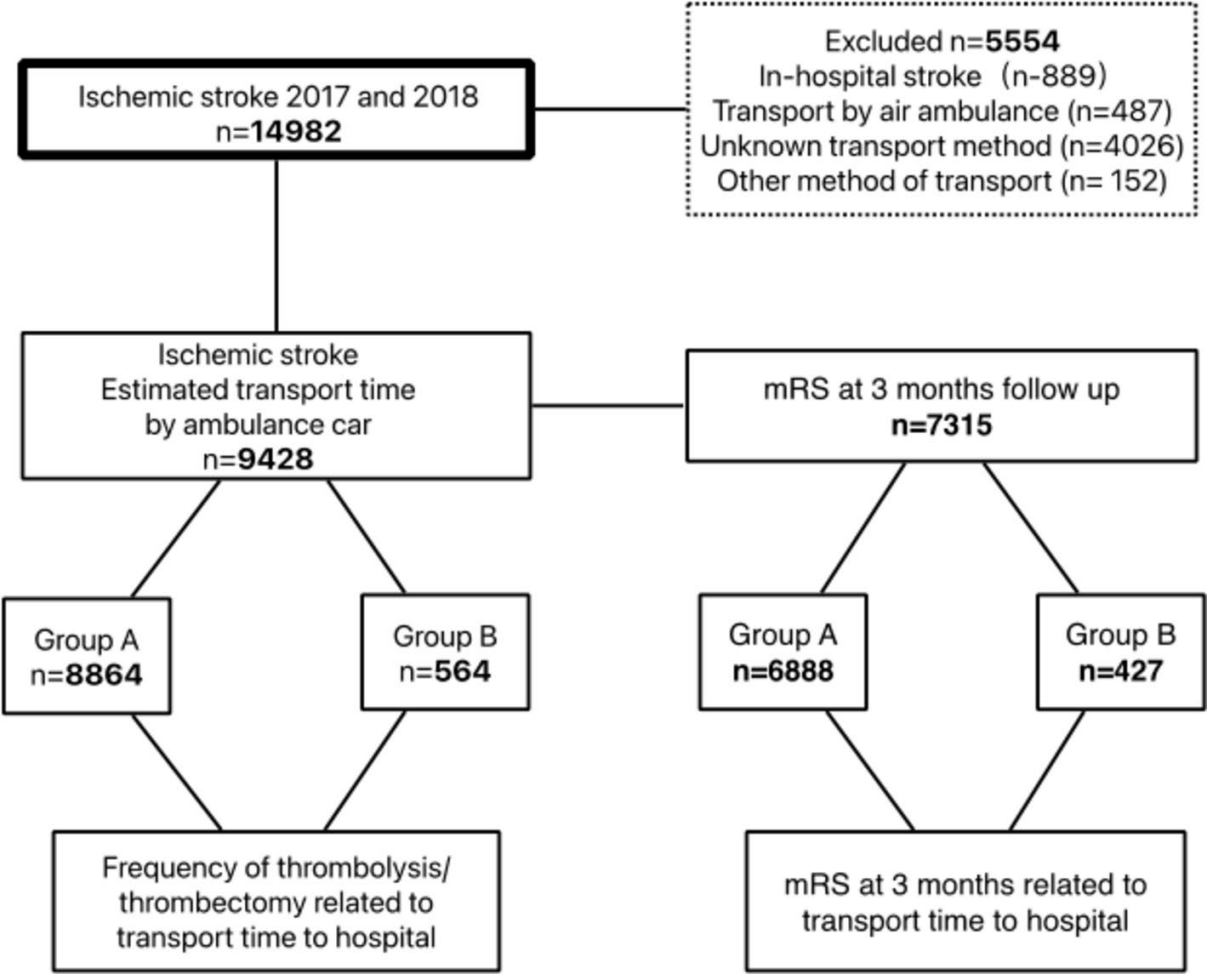
Flowchart of data collection and the division in patient groups. mRS: modified Rankin Scale

All hospitals treating patients with stroke (ICD-10 diagnosis I61; Hemorrhagic Stroke, I63; Ischemic Stroke and I64; Unspecified Stroke) are obliged to enter their cases into the Norwegian Stroke Registry (NSR) [14]. We collectively analyzed all strokes registered in the years 2017 and 2018. We included only patients with ischemic stroke aiming to investigate the frequency of reperfusion therapy related to transport time. Patients suffering from stroke while hospitalized or who were transported by air ambulance or had unknown/other method of transport, were excluded from study, Fig. 1. Clinical characteristics were reported as registered in the NSR [15].

Patients with ischemic stroke were categorized into two groups based on their estimated transport time from home to the nearest hospital offering acute thrombolytic treatment. Patients in Group A were defined as having an estimated transport time of less than 63 min. Patients designated to Group B had an estimated transport time of more than 63 min, Fig. 1. The time limit of 63 min was chosen taking estimations of the other various time components of onset of symptoms to treatment and the nationally recommended 180 min time limit [5] for thrombolytic treatment into consideration as follows (median times, source in parentheses): Onset of symptoms to alarm; 34 min (unpublished registry data from NSR), alarm to arrival of ambulance in rural municipalities; 31 min (data from the Norwegian Directorate of Health [16]), ambulance on scene time; 21 min (data from a prehospital study in Denmark [17]) and door to needle time; 31 min (registry data from NSR [15]). We applied the sum of these medians (117 min) as an overall estimate of delay not being due to time in transport. Thus, patients having an estimated transport time of less than 63 min (180 min–117 min) would according to the above have an onset of symptoms to treatment time within the recommended time limit for thrombolysis.

In cooperation with the national statistical institute, Statistics Norway, we linked the national identity number of each individual to the Norwegian National Registry thus obtaining the patients´ home addresses at the time of stroke ictus. The addresses of the patients and the nearest hospital offering acute thrombolytic treatment then formed the basis for calculation of driving time from home to hospital using the National Road Database according to the DCAT-AP-NO standard [12]. Transportation time in ambulance was set to 80% of the calculated driving time at speed limits [18]. Ferry transport times were determined by distance over sea in 15 km/h plus 10 min for boarding and exit.

### Statistical analysis

Clinical characteristics of the two groups were compared by Chi squared test for categorical and Mann-Whitney U test for continuous variables. We related the frequency of thrombolysis to transport time by logistic regression analysis. The administration of thrombolysis and thrombectomy was compared by Chi squared test. Predictors of thrombolysis other than transport time were analyzed by multivariate logistic regression. Time from onset of symptoms to bolus dose of thrombolysis as well as modified Rankin Scale (mRS) at 3-month follow-up for the two groups were compared by Mann-Whitney U test. A multivariate regression analysis was performed to explore the relationship between clinical outcome, time in transport and clinical characteristics between the two groups. Significance level was set to *p* < 0.01. We applied the statistical software IBM SPSS Statistics Version 28 and STATA 18 for all analyses.

### Ethical considerations

We collected data from NSR and Statistics Norway after obtaining permission from the Regional Ethical Committee (2019/1260), the Norwegian Institute of Public Health (19/11988) and the local Data Protection Officer in Vestre Viken Hospital Trust.

## Results

For the years 2017 and 2018, 51 hospitals reported a total of 17,629 stroke cases and this represented 87% of all strokes recorded in hospital journal systems. A 3-month follow-up registration was completed in 77% of the registered patients. A total of 14,982 patients were registered as having suffered an ischemic stroke, Fig. 1. Of these 9428 (62,9%) were reported as transported in ambulance while 5554 (37,1%) had non-ambulance transport. Among ambulance transported patients 8864 (94.0%) patients were categorized into Group A, whereas 564 patients (6.0%) had, according to our definition, prolonged transport, Group B. The clinical characteristics of these groups are listed and compared in Table 1.

**Table 1:** Main characteristics and outcomes of patients with ischemic stroke

	Total patients		*P*-value	Ambulance patients		*P*-value
	Ambulance	Non-ambulance		Group A	Group B	
Index stroke 2017/18 – no.	9428 (62.9)	5554 (37.1)		8864 (94.0)	564 (6.0)	
Age, mean (SD)	75.6 (12.9)	71.4 (12.9)	<0.05	75.6 (12.9)	75.3 (12.2)	0.29
Sex, female (%)	4402 (46.7)	2272 (40.9)	<0.05	4146 (46.8)	256 (45.4)	0.52
Prestroke mRS^a^, mean (SD)	1.1 (1.3)	0.6 (1.0)	<0.05	1.0 (1.3)	1.1 (1.2)	0.15
0 - 2 – no. (%)	6913 (82.0)	4640 (92.8)	<0.05	6487 (81.8)	426 (84.7)	0.11
Medical history						
Previous stroke – no. (%)	2333 (24.7)	1058 (19.0)	<0.05	2195 (24.8)	138 (24.5)	0.96
Previous TIA – no. (%)	1028 (10.9)	550 (9.9)	0.28	957 (10.8)	7.1 (12.6)	0.12
Atrial fibrillation/flutter – no. (%).	2609 (27.7)	1195 (21.5)	<0.05	2438 (27.5)	171 (30.3)	0.23
Hypertension, medically treated – no. (%)	5649 (59.9)	3117 (56.1)	<0.05	5279 (59.6)	370 (65.6)	<0.05
Myocardial infarction – no. (%)	1425 (15.1)	764 (13.8)	0.46	1323 (14.9)	102 (18.1)	0.10
Diabetes Mellitus – no. (%)	1837 (19.5)	1108 (19.9)	0.74	1723 (19.4)	114 (20.2)	0.21
Current smoking – no. (%)	1777 (18.8)	1258 (22.7)	<0.05	1662 (18.8)	115 (20.4)	<0.05
Wake up stroke^b^	1878 (23.7)	1080 (25.5)	0.31	1751 (23.5)	127 (27.1)	0.71
NIHSS^c^ at hospitalization, median (IQR)	4 (1–7)	2 (1–4)	<0.05	4 (2–9)	3 (1–7)	0.09
0 - 5 – no. (%)	4934 (61.8)	3300 (81.9)	<0.05	4 651 (61.5)	283 (66.3)	<0.05
6 - 14 – no. (%)	2034 (25.5)	492 (12.2)	<0.05	1931 (25.5)	103 (24.1)	0.29
≥ 15 – no. (%)	1017 (12.7)	238 (5.9)	<0.05	976 (12.9)	41 (9.6)	0.95
First admission to stroke unit – no. (%)	7792 (82.6)	4259 (76.7)	<0.05	7366 (83.1)	491 (87.1)	<0.05
Referred for rehabilitation^d^ – no. (%)	2270 (24.1)	069 (19.2)	<0.05	2142 (24.2)	128 (22.7)	0.49
mRS at 3-month follow-up, mean (SD)	2.6 (2.1)	1,9 (1,8)	<0.05	2.6 (2.1)	2.6 (2.0)	0.99
0 - 2 – no. (%)	4203 (57.5)	3172 (73.5)	<0.05	3957 (57.4)	246 (57.6)	0.95

**Total patients:** Ischemic stroke patients 2017 and 2018 registered in NSR

**Ambulance patients:** Ischemic stroke patients with known transport in ambulance

**Non-ambulance patients:** Ischemic stroke patients with unknown or other transport method

**Group A:** Ambulance patients with transport time < 63 minutes

**Group B:** Ambulance patients with transport time > 63 minutes

^a^*mRS* modified Rankin Scale

^b^ Stroke symptoms upon awakening

^c^*NIHSS* National Institute of Health Stroke Scale

^d^ Include both specialized and municipal rehabilitation

Compared to patients with non-ambulance transport, patients transported by ambulance had a poorer premorbid status, a reduced frequency of minor strokes (NIHSS 0–5) and a worse outcome as judged by mRS at 3-months follow-up. Among the ambulance transported patients, the group with prolonged transport, Group B, had a higher prevalence of hypertension and current smoking than Group A. They also had a slightly higher frequency of minor strokes and were to a greater extent admitted directly to a stroke unit when hospitalized.

### Transport time

**Fig. 2:**
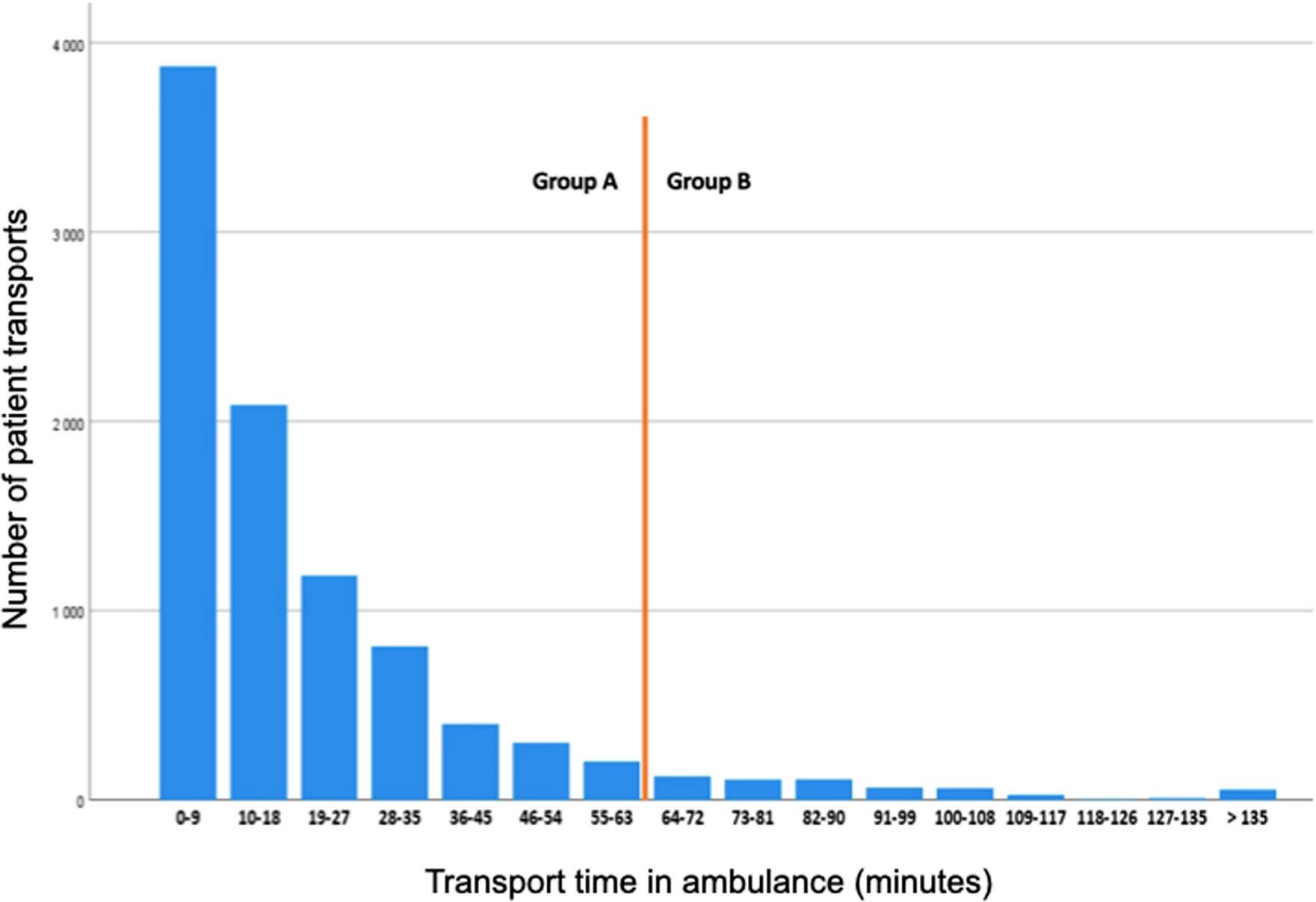
Distribution of estimated transport times in ambulance from home to hospital for patients with ischemic stroke 2017 and 2018. The red vertical line illustrates the division of the two patient categories

The distribution of estimated transport times in ambulance for patients with ischemic stroke was highly skewed to the left with a mean of 20.4 min (SD 24.0) and a median of 11.9 min (IQR 5.3–26.4), Fig. 2.

### Frequency of thrombolysis

**Fig. 3:**
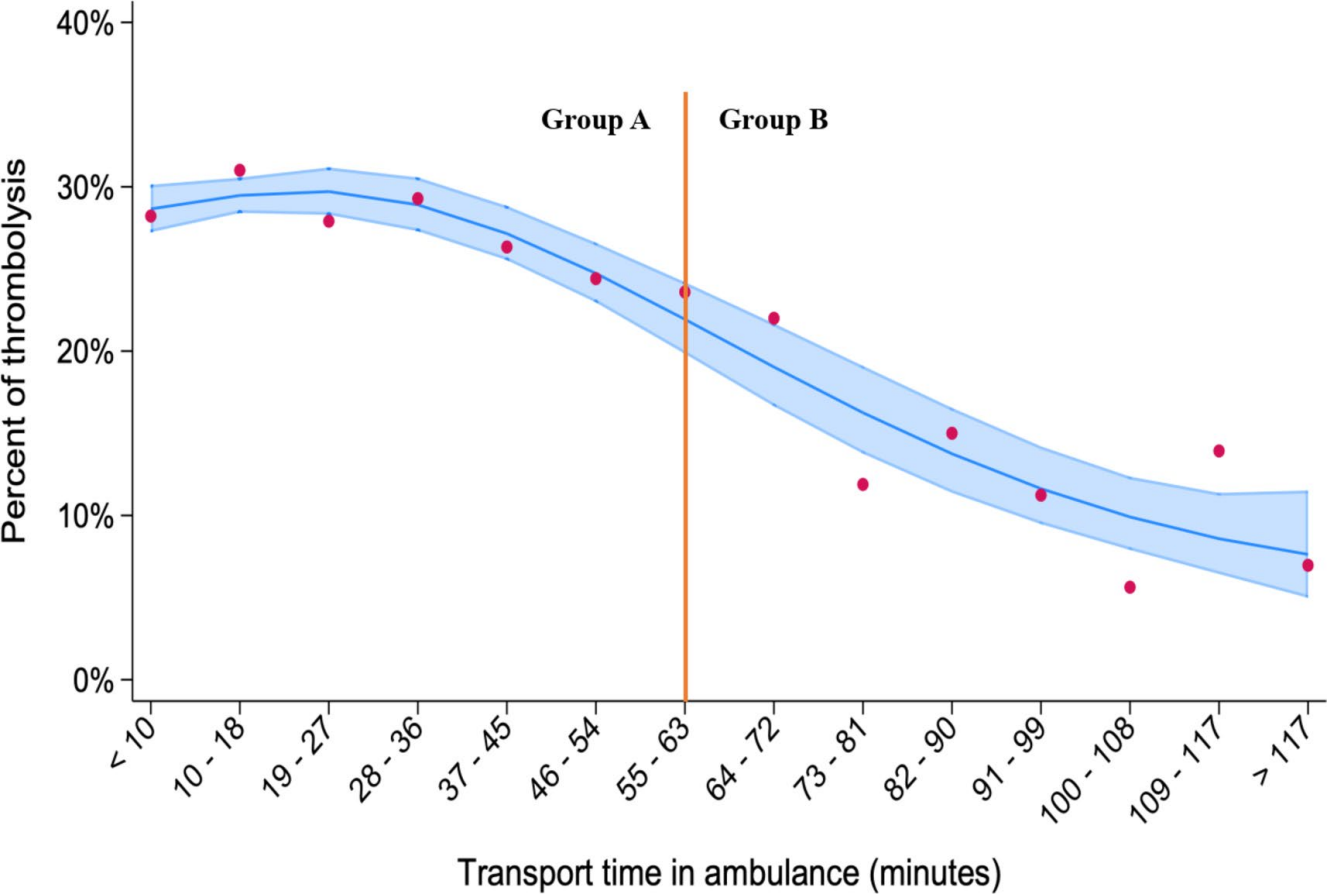
Plot of percent receiving thrombolysis within categories of transport time in ambulance for patients with ischemic stroke. The red vertical line illustrates the divide between the two patient groups. Spline curve for trend (*p* < 0.01) with 95% CI

As illustrated in Fig. 3, the frequency of thrombolytic treatment decreased with increasing time spent in ambulance transport to hospital.

In Group A, 2499 of 8 864 patients received intravenous thrombolysis (28.2%) versus 59 out of 564 patients (10.5%) in Group B, (OR 95% CI) 2.68 [2.26–3.21], *p* < 0.01. For patients treated with thrombolysis the median (IQR) time from onset of symptoms to bolus dose of alteplase was 110 min (69–152) in Group A versus 180 min (139–222) in Group B, *p* < 0.01. In multivariate logistic regression, in which the 14 transport time categories displayed in Fig. 3 were entered together with age, sex and clinical characteristics showing between groups significant differences in univariate analysis (Table 1), we found that (OR, 95% CI) hypertension 1.19 [1.11–1.27], increasing NIHSS score 1,05 [1.04–1.06] and transport time category 0.93 [0.91–0.95] were independently related to thrombolytic treatment. Thrombectomy was performed in 431 patients (4.9%) in Group A versus 12 patients (2.1%) in Group B, *p* < 0.01.

### Clinical outcome

**Fig. 4:**
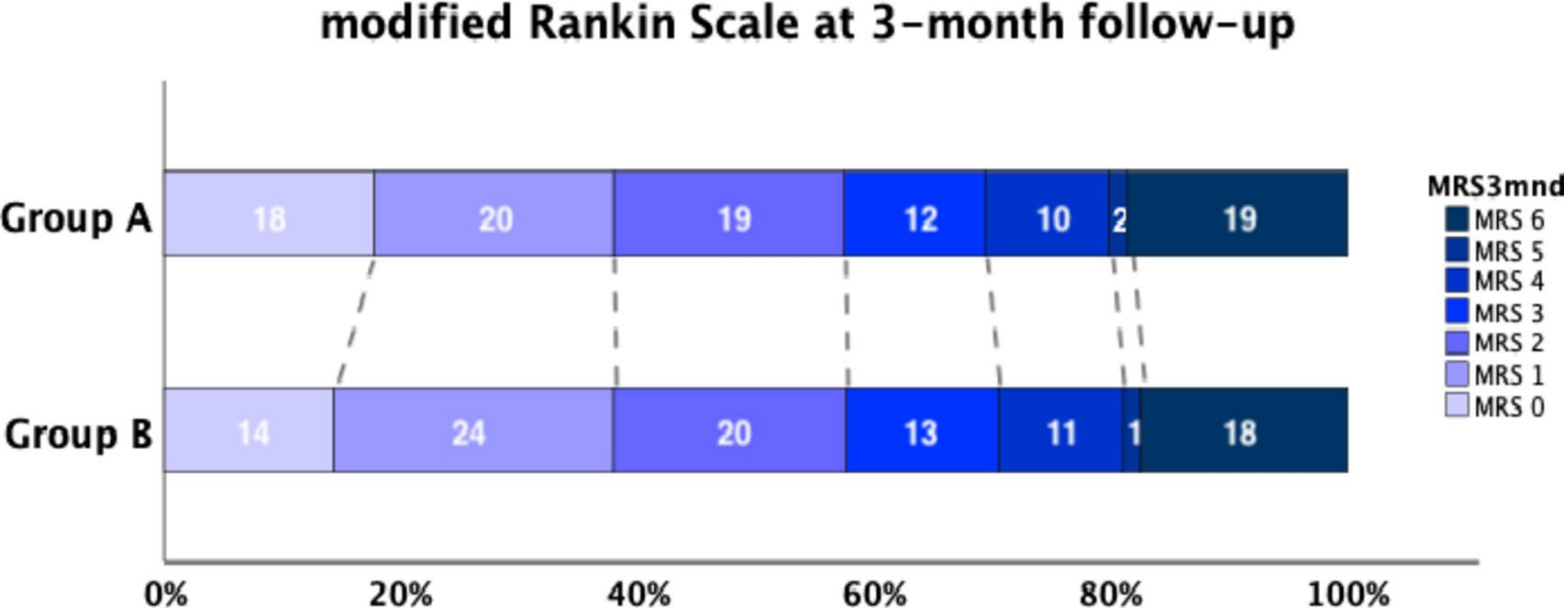
Distribution of modified rankin scale at 3-month follow-up in the two groups

Three months follow up was completed in 7315 ambulance transported patients with ischemic stroke. As depicted in Fig. 1, 6888 patients were classified into Group A and 427 into Group B. Clinical outcome, as judged by the mRS at 3-month follow-up, is displayed in Fig. 4. 57% of patients in Group A and 58% in Group B had a good outcome (mRS 0–2) (OR 95% CI) 0.95 [0.81–1.12], *p* = 0.57. In a multivariate logistic regression analysis of the follow-up dataset the 14 transport time categories displayed in Fig. 3 were entered together with age, sex and clinical characteristics showing between groups significant differences in univariate analysis (Table 1). We found that that only low age and low NIHSS score increased the probability for good outcome with (OR 95% CI) 1.08 [1.07–1.09] and 1.19 [1.17–1.20] respectively. The percent of patients in Group A and Group B with mRS 6 (dead) were 19% vs. 18%, (OR 95% CI) 1.04 [0,85–1.28], *p* = 0.72. In multivariate analysis (OR 95% CI) low age 0.92 [0.91–0.93] and low NIHSS score 0.87 [0.86–0.88] reduced the probability for death. There was no statistically significant difference between the groups with regard to overall mRS distribution, *p* = 0.82.

## Discussion

In the present study we found that the frequency of thrombolysis showed a downward trend as transport time in ambulance to hospital increased. The rate of thrombolysis was significantly higher in in patients living in areas without prolonged transport. Also, the timing of thrombolytic treatment when given was significantly earlier in this group than for patients with a prolonged transport (median of 110 versus 180 min). We could not however demonstrate a difference between the groups in 3-month outcome as judged by the mRS.

Due to the time constraints of thrombolytic treatment effect, our finding of a significant inverse relationship between transport time and administration of thrombolysis was as expected. Surprisingly, although the topography of Norway offers clear challenges for road building and transport, only 6% of ambulance transported patients had what we defined as a clinically relevant prolonged transport time (63 min and above). This number is an estimate only. All time components of the stroke chain were not available on an individual basis and we chose to utilize reported time medians to estimate the critical 63 min limit of transport. As seen in Fig. 3, estimated transport time seemed to impact the frequency of thrombolysis also below this limit. Nevertheless, if our findings reflect the real-world situation, it appears that long ambulance transport times may constitute a relatively minor problem in acute stroke care for the population as a whole.

While stroke patients with prolonged transport seemed to be at a disadvantage with regard to access to thrombolysis and thrombectomy, other factors than transport time contributed to this. The groups differed in clinical characteristics as relatively more patients with prolonged transport had hypertension and to a greater extent suffered from minor strokes. While the explanation for these findings remain unclear, these factors in multivariate analysis also significantly decreased the chance of receiving thrombolysis. Therefore, in addition to the time aspect, clinical factors put the group of patients with prolonged transport at a disadvantage for receiving thrombolytic therapy. Possibly, other non-recorded factors such as differences in stroke awareness between rural and urban populations, may have impacted the results.

The inverse relationship between transport time and frequency of thrombolysis was previously reported in a study from South Australia [19]. The authors found lower rates in the population living furthest away from hospital. Also, a study from the U.S. demonstrated that rural patients with stroke to a lesser extent were treated by intravenous thrombolysis or endovascular therapy and had higher mortality in hospital than patients from urban areas [20].

While frequency of thrombolysis and thrombectomy were significantly lower for patients with prolonged transport, we could not demonstrate a worsened 3-month outcome for this group as expressed by the mRS. We see several reasons for this: There was a relatively low frequency of thrombolysis in the two groups (28.2% versus 10.5%) which were highly unbalanced with a low number of patients in the latter group (*n* = 427). This limited the statistical power for showing a difference on the ordinal mRS. There were significant clinical differences between the two groups apart from time in transport that may have influenced the clinical outcome. Also, other non-recorded variables may have confounded the analyses. Finally, our estimations of transport times and the other time components of the stroke chain were a source of imprecision.

Thrombectomy has a broader therapeutic time window than thrombolysis and is the preferred treatment of Large Vessel Occlusion stroke [21]. Still the frequency of thrombectomy in patients with prolonged transport was significantly reduced (4.9% versus 2.1%). A slightly higher number of minor strokes in the prolonged transport group might have affected this result. In the studied period, thrombectomy was still in its clinical implementation phase. Long and cumbersome transports to a CSC as well as a low case-frequency may at the time have slowed the development of effective thrombectomy routines in the rural PSCs.

The Norwegian Emergency Medical Service is regionally administered by each Hospital Trust. A nationwide protocol is utilized for recognition of stroke symptoms and dispatch of ambulance [5]. However, as illustrated in Fig. 1, there were more than 4000 patients with unknown or other method of transport and these patients were excluded from analysis. While these missing observations represents a limiting factor for our analyses we found that estimated median (IQR) transport time for this subgroup was 13 min (1–25) which is comparable to the group registered as transported in ambulance (12 min (5–26)), pointing to a similar geographical distribution. In the group with unknown or other method of transport we found significantly more patients with lower pre-stroke mRS and more minor strokes compared to the group transported by ambulance. This result corresponds to previous studies showing that patients with minor strokes to a lesser extent have ambulance transport [11].

Transport for ischemic stroke patients consumed more than 35% of the estimated total prehospital delay in the group of patients with prolonged transport. Time spent in transport by ambulance car is dependent on road standard. While road standard is steadily albeit slowly improving, it will probably have minor impact on national transport times. Transport of stroke patients by helicopter emergency medical services (HEMS) occurs in less than 5% of cases (NSR 2017, unpublished). HEMS have limited resources and other acute cases contribute to contemporary conflicts [22].

Alternatives for moving acute diagnosis and treatment out of hospital and closer to the patient are currently explored. Mobile Stroke Units are tested in many countries and the results are encouraging [23, 24]. In Norway we have since 2017 operated a CT station and thrombolytic service at a local district medical centre in the rural area of Hallingdal [25]. As recently published we found this model to be clinically feasible as well as time saving with regard to transport [26].

### Study limitations

Firstly, in our analyses we assumed that all strokes took place at patient home. Data from the Framingham study (1995) showed that 10% of ischemic strokes occurred outside of patient’s home (in-hospital strokes excluded) [27]. We have not found more recent data that could shed light on out of home stroke frequency. Secondly, each health region administers its own Emergency Medical System and the various time components of the acute stroke chain may vary considerably. In addition, some patients may have been transported without the “lights and sirens” routine. Thus, the relative importance of transport time as a limiting factor may be highly variable across districts. Unfortunately, we did not have access to individual data of other stroke chain time components. The applied approximations will consequently not accurately reflect real world data. Furthermore, specific information about why iv tPA was not given in the individual patient was lacking. Lastly, imbalances in unreported baseline characteristics between the two groups may have influenced the results. We report historical data for 2017 and 2018. However, present day the frequency of reperfusion therapy has increased only slightly since then (2018: thrombolysis; 21%, thrombectomy; 5%, 2023: thrombolysis; 22%, thrombectomy; 6% [1, 16]. This minimal progression indicates a continued prehospital delay.

In conclusion, we found that based on the National Stroke Registry for 2017 and 2018 only 6% of Norwegian stroke patients lived in areas defined by an estimated ambulance transport time of more than 63 min. With increasing time spent in transport, a lower rate of reperfusion therapy was observed. The relatively low number of patients affected by long transport time did not permit a meaningful analysis of a possible worsened outcome for the group. All components in the chain of stroke survival should be continuously optimized for more rapid stroke care. Prehospital alternatives for diagnostic work up and treatment seem especially important for the patients living in rural areas far away from the nearest CT equipped hospital.

## Data Availability

No datasets were generated or analysed during the current study.
